# Incorporating a Professional-Grade All-Class Project Into a Research Methods Course

**DOI:** 10.3389/fpsyg.2018.02143

**Published:** 2018-11-06

**Authors:** Frank M. LoSchiavo

**Affiliations:** Department of Psychology, Ohio University Zanesville, Zanesville, OH, United States

**Keywords:** research methods, undergraduate research, publishable research, group research projects, best practices in teaching

## Introduction

Because psychology is a young and ever-expanding science, research methods courses play a particularly important role in the undergraduate curriculum. For example, the American Psychological Association (APA, 2013) recommends that undergraduate psychology programs require students to complete research methods courses early so that advanced courses can build upon a commonly shared understanding of empirical psychology. Research methods courses also provide most students with their first opportunity to collaborate with their professors and peers on data-collection projects. Thus, the tone set by a research methods course is critically important, because more so than any other class, it will color the way students view psychological science. It is during that initial research methods course that we should set our collective expectations high and require undergraduate students to conduct professional-grade, potentially publishable research projects.

That said, faculty cannot demand potentially publishable research from their novice students without offering them sufficient instructional support. The teaching model I have developed provides effective support and incorporates best practices designed to maximize the probability of producing publishable research. This new model abandons traditional teaching techniques that focus primarily on daily lectures, classroom demonstrations, and brief research projects, because those techniques may oversimplify psychological science and may unintentionally teach students that they are not yet ready to publish their own professional-quality research.

## Professional-grade all-class project

My research methods courses focus on conducting group research projects of my choosing. For example, a previous class immersed themselves in a semester-long laboratory experiment designed to test the dubious claim that specialized software (i.e., Truster, 1997) could detect deception via voice stress analysis (Meyer, 1998; Van Damme, 2001; Taylor, 2002; for a review, see Lykken, 1998). Although the class read a brief (350-page) introductory research methods textbook (i.e., Martin, 2000) and was tested on its content via a midterm examination, they spent most of their time reviewing the literature on deception, designing the study, collecting data, analyzing results, and writing APA-style manuscripts. That project debunked the now-defunct software manufacturer's surprisingly positive claims and led to a published article coauthored by an undergraduate student (LoSchiavo and Roberts, 2005).

Although research methods courses are tough to teach, weaving an entire course around a single, professional-grade, group project can make the task more manageable. For example, when an entire class collaborates on a single project, data-collection efforts are more efficient and result in larger samples. Such efficiencies allow the students and the instructor to focus on mastering the topic of investigation and on the necessary methods to be used. And because professional research is often programmatic, results from one study point student investigators to the need for additional, but related, research. Thus, results obtained by current students can replicate results conducted by previous students and inform research conducted by future students. This continuity can provide the course with more coherence. Furthermore, as future students refine hypotheses and improve upon their predecessors' methods, results will more likely support predictions, and consequently, research projects will become more publishable.

## Best practices for designing the course

Lessons learned over time have helped me develop several best practices that guide current course projects. The basic guidelines that follow allow instructors to cover a great deal of ground within a single semester.

## Guideline 1: negotiate departmental support

Although some faculty have complete autonomy over how they structure their courses, many do not, typically because departmental policies standardize specific courses, or because departments mandate that particular course objectives be met by all instructors. Thus, some faculty may need to argue the merits of conducting professional-grade projects while negotiating variances from standard departmental procedures.

Furthermore, some departments may not possess the requisite equipment or infrastructure for a particular research study. For example, when a campus building project left my department without laboratory space for a class study on extrasensory perception (ESP), we successfully negotiated use of soundproof practice rooms in the music department.

## Guideline 2: select projects that maximize interest and publishability

I initially selected course projects designed to test pseudoscientific claims (e.g., communication via ESP), but it became clear that students were less excited by studies predicting null results, which often remain inconclusive. Now I select projects that focus on emerging topics that prove interesting and publishable regardless of the outcome. For example, a recent class tested whether thermal imaging could distinguish between liars and truth-tellers during an interrogation that followed a simulated theft. Previously published results suggest that thermal imaging might be useful for detecting deception (e.g., Pavlidis et al., 2002; Warmelink et al., 2011), but the technology's usefulness is constrained by many factors, so virtually all new data is publishable at this time.

It is also wise to select projects that students can master quickly, both conceptually and methodologically. Topics in applied psychology make ideal candidates. Research on detecting deception, for example, is relatively straightforward, yet conceptually relevant to psychology, and it offers a host of methodologies that novice researchers can employ (e.g., simulated thefts and concealed information tests).

## Guideline 3: complete pre-semester preparatory work

My teaching model requires considerable preparatory work prior to the start of the semester. For example, if the project requires institutional review, instructors should submit a proposal before the course begins, because review boards are slow to grant approval. In addition, instructors should conduct a literature review and create a local information archive comprised of key books and articles that students may want to read once they have been tasked with reviewing the literature themselves. This local archive will allow students to obtain sources quickly, without having to suffer the delays of interlibrary loans. It will also eliminate the possibility of some students hoarding sources that other students would like to borrow.

## Guideline 4: develop a basic course calendar

If instructors hope to teach the basics of research methods and to conduct a professional-grade, group research project within one 15-week semester, then it is critically important for them to create an efficient course calendar. In the teaching model that I have developed, students spend the first 4 weeks of the semester plowing through a brief research methods textbook (e.g., Patten, 2014). The book is designed to provide an overview of the essential concepts typically covered in a research methods course. Although I support their readings with short lectures, students spend considerable class time during these first few weeks reviewing the project literature and completing online research-ethics training, which is mandatory at our university. Then, during the fifth week, students complete a midterm examination covering the basic concepts discussed in class and in the textbook.

The fun begins in the sixth and seventh weeks, when students design the study, practice the laboratory procedures, and then pilot test a small group of participants during week eight. By the ninth week, data collection is well underway, with students taking laboratory shifts as their schedules permit. During the twelfth and thirteenth weeks, the class meets to analyze the data and discuss strategies for writing research reports. Then, during the fourteenth and fifteenth weeks of the semester, groups of 2–3 students write APA-style manuscripts chronicling the entire project that they submit for a grade instead of completing a final examination. Although students form their own groups and write much of the manuscript outside of class, all students meet in class during the final few weeks of the semester to exchange ideas and to seek my feedback on what they have written so far.

Depending on the results of the study and the quality of the manuscripts, I may (or may not) encourage particularly talented groups to collaborate on a multiauthor final manuscript that can be submitted for publication, knowing that some students might find the challenge exciting, while others might not be interested in additional work. Students who accept the challenge must determine who will be responsible for various sections of the manuscript, and they must agree on authorship issues, with the understanding that the entire class will be credited in a footnote.

## Guideline 5: exploit time efficiencies

Although creating a basic course calendar is a good start, a simple timeline is often too unidimensional to capture the nuances behind successful time management. To complete so much work in one semester, instructors must constantly search for ways to make efficient use of time, and that usually involves planning specific activities a few weeks in advance. For example, when students conduct literature reviews during week two, I assign key articles for summarization so that students will be familiar with basic findings and fundamental methods when it is time to design the research project in week six. Likewise, when students create skeleton SPSS data files during week eleven, we spend class time declaring hypotheses so that they are in place before entering and analyzing data in week thirteen.

These subtle efficiencies can save considerable class time. They can also help students organize their thoughts as they complete complex assignments. For example, when students create SPSS syntax files during week twelve, we order the programming code for each analysis so that it coincides with where we estimate results will be reported in the manuscript. This preparation helps students during the final weeks of the semester, as they work in small groups writing APA-style research reports. Thus, it is important to understand that each component of the course calendar is interconnected, and that instructors can prime future topics by introducing key aspects of those topics several weeks in advance.

## Guideline 6: do not overestimate your students' abilities

Success using the teaching model I have proposed depends upon estimating students' abilities accurately, and as I mentioned previously, research methods courses provide most students with their first opportunity to collaborate on data-collection projects. In other words, these students are novice researchers. Without necessary leadership, their projects will likely fail, often because students lack the experience necessary to see hurdles hidden far down the road. Thus, to complete a research project worthy of publication within a single semester, instructors may need to make key methodological decisions, and they will be required to do significant work in between classes. For example, after my students brainstorm general ideas in class, I often find myself working on the details in my office. Based on student input, I have created comprehensive laboratory manuals (which have included step-by-step methodological instructions), survey instruments, and other project-related materials. Furthermore, I often spend considerable time recruiting participants from other classes. In fact, I have spent so much time recruiting participants that some days I have felt less like an instructor and more like a laboratory assistant.

## Guideline 7: do not underestimate your students' abilities

With so much to accomplish in one semester, the preceding guidelines have focused on how instructors can structure research methods courses to maximize efficiency and productivity. Although instructors should make key decisions while serving as class leaders, they should avoid underestimating their students' abilities, and they should allow students as much autonomy as possible. I have always been impressed with how creative students can be in finding solutions both to small problems that occur daily and to large methodological issues that might otherwise stall an entire project. For example, while designing a class project on ESP, my students developed an elaborate knocking procedure that allowed them to communicate between themselves while research participants thought they were simply knocking to enter a room.

## Concluding remarks

As a discipline, we expect our graduate students to publish their research, and we have built an elaborate pedagogical system that encourages it. I suggest that we consider expecting a bit more from our undergraduate students, as well. But first, we need to make changes to how we typically teach introductory research methods courses. By focusing on professional-grade, potentially publishable research projects, my students have developed a better understanding of the steps necessary to produce published research of their own.

## Author contributions

FL confirms being the sole contributor of this work.

### Conflict of interest statement

The author declares that the research was conducted in the absence of any commercial or financial relationships that could be construed as a potential conflict of interest.
